# Remote monitoring data from cardiac implantable electronic devices predicts all-cause mortality

**DOI:** 10.1093/europace/euab160

**Published:** 2021-10-03

**Authors:** Fozia Zahir Ahmed, Camilla Sammut-Powell, Chun Shing Kwok, Tricia Tay, Manish Motwani, Glen P Martin, Joanne K Taylor

**Affiliations:** Division of Cardiovascular Sciences, Faculty of Biology, Medicine and Health, University of Manchester, Manchester, UK; Department of Cardiology, Manchester University Hospitals NHS Foundation Trust, Oxford Rd, Manchester, UK; Division of Informatics, Imaging and Data Sciences, Faculty of Biology, Medicine and Health, University of Manchester, Manchester Academic Health Science Centre, Manchester, UK; School of Primary, Community and Social Care, Keele University, Stoke-on-Trent, UK; Department of Cardiology, University Hospitals of North Midlands NHS Trust, Stoke-on-Trent, UK; Division of Cardiovascular Sciences, Faculty of Biology, Medicine and Health, University of Manchester, Manchester, UK; Division of Cardiovascular Sciences, Faculty of Biology, Medicine and Health, University of Manchester, Manchester, UK; Department of Cardiology, Manchester University Hospitals NHS Foundation Trust, Oxford Rd, Manchester, UK; Division of Informatics, Imaging and Data Sciences, Faculty of Biology, Medicine and Health, University of Manchester, Manchester Academic Health Science Centre, Manchester, UK; Division of Informatics, Imaging and Data Sciences, Faculty of Biology, Medicine and Health, University of Manchester, Manchester Academic Health Science Centre, Manchester, UK

**Keywords:** Cardiac resynchronization, Defibrillators, Prognosis, Risk score, Mortality, Remote monitoring

## Abstract

**Aims:**

To determine if remotely monitored physiological data from cardiac implantable electronic devices (CIEDs) can be used to identify patients at high risk of mortality.

**Methods and results:**

This study evaluated whether a risk score based on CIED physiological data (Triage-Heart Failure Risk Status, ‘Triage-HFRS’, previously validated to predict heart failure (HF) events) can identify patients at high risk of death. Four hundred and thirty-nine adults with CIEDs were prospectively enrolled. Primary observed outcome was all-cause mortality (median follow-up: 702 days). Several physiological parameters [including heart rate profile, atrial fibrillation/tachycardia (AF/AT) burden, ventricular rate during AT/AF, physical activity, thoracic impedance, therapies for ventricular tachycardia/fibrillation] were continuously monitored by CIEDs and dynamically combined to produce a Triage-HFRS every 24 h. According to transmissions patients were categorized into ‘high-risk’ or ‘never high-risk’ groups. During follow-up, 285 patients (65%) had a high-risk episode and 60 patients (14%) died (50 in high-risk group; 10 in never high-risk group). Significantly more cardiovascular deaths were observed in the high-risk group, with mortality rates across groups of high vs. never-high 10.3% vs. <4.0%; *P* = 0.03. Experiencing any high-risk episode was associated with a substantially increased risk of death [odds ratio (OR): 3.07, 95% confidence interval (CI): 1.57–6.58, *P* = 0.002]. Furthermore, each high-risk episode ≥14 consecutive days was associated with increased odds of death (OR: 1.26, 95% CI: 1.06–1.48; *P* = 0.006).

**Conclusion:**

Remote monitoring data from CIEDs can be used to identify patients at higher risk of all-cause mortality as well as HF events. Distinct from other prognostic scores, this approach is automated and continuously updated.

**What’s new?**

- Modern cardiac implantable electronic devices (CIEDs) include multiple sensors which facilitate continuous data collection of monitored physiological parameters. These parameters can then be dynamically combined to produce prognostic metrics, for example the validated Triage-Heart Failure Risk Status ‘Triage HFRS’.
- This is the first prospective study to report that HFRS data, derived from remotely monitored CIED data and originally developed to identify patients at increased risk of heart failure hospitalization, can be used to predict all-cause mortality.
- Routinely monitored data, automatically collected on a daily basis can help discriminate between ambulatory patients at high and low risk of death, i.e. stratify risk remotely.
- This study reports a three-fold increased odds of mortality for patients who spent at least 1 day in a high HFRS status, and a 26% increase in odds of mortality for patients who had 14 consecutive days or more in a high-risk status.
- Higher percentages of time spent in a ‘high’ risk status, and less time in a ‘low’ risk status, were associated with increased risk of death.

## Introduction

Patients who have cardiac implantable electronic devices (CIEDs) due to underlying heart disease are a heterogeneous cohort with varying degrees of morbidity, mortality risk, and healthcare utilization. Modern CIEDs have the benefit of remote monitoring capabilities which enable detection of real-time physiological parameters of patients in their own homes. The remotely captured data can help identify episodes of decompensation as they occur and also help predict future adverse clinical events.^1–4^ Utilization of this rapidly advancing technology for ‘real-time’ risk stratification could provide a paradigm shift in the management of cardiac patients, particularly those with heart failure (HF).

Predicting mortality in an ambulatory CIED population is notoriously challenging for a variety of reasons. First, rhythm problems rarely exist in isolation, especially in older patients who have varying degrees of comorbidity and frailty. Second, a high proportion of patients with CIEDs have HF, which classically follows a relapsing/remitting course. Third, HF has multiple aetiologies which can give rise to different risk profiles. Finally, existing risk prediction tools used in patients with CIEDs and those with HF are limited in that they require knowledge of directly observed parameters (e.g. blood pressure, height, weight, functional class, ejection fraction, creatinine and medication) which are not always available.^5–9^ Therefore, the concept of utilizing CIEDs for remote monitoring is ideal because several parameters that correlate with heart function stability [e.g. thoracic impedance, presence of tachycardia, atrial arrhythmias, percentage of cardiac resynchronization therapy (CRT) pacing, activity levels etc.] are measured automatically by the devices in real-time.

The validated ‘Triage Heart Failure Risk Status’ (Triage-HFRS) is a risk prediction model, which uses the physiological parameters collected from Medtronic CIEDs to risk-stratify patients as low-, medium-, or high risk of HF events within 30 days.^2^ The risk status algorithm considers a combination of up to nine parameters depending on the device features (atrial tachycardia ‘AF’ / atrial fibrillation ‘AF’ duration, ventricular rate during AT/AF, OptiVol™ fluid index, patient activity, night heart rate, heart rate variability (HRV), percentage of CRT pacing, treated ventricular tachycardia/ventricular fibrillation, and defibrillator shocks). Triage-HFRS was originally designed to predict HF hospitalization events, i.e. hospitalization due to HF decompensation. Prior studies have shown that Triage-HFRS has high sensitivity for predicting worsening HF events, but no study has prospectively explored whether it predicts all-cause mortality.^2^^,^^3^ This is important, as anticipated life expectancy impacts upon clinical decision-making above and beyond short-term risk of disease instability.

The aim of this study was to investigate if device-derived physiological data (summarized as sensor data used by the Triage-HFRS) can predict risk of all-cause mortality. Secondly, we investigated if the addition of easily obtained demographic variables altered this association.

## Methods

### Study design, setting, and participants

This study was a prospective, single-site observational study undertaken in accordance with the STROBE statement for reporting observational studies.^10^

The Manchester Heart Centre (MHC) is a tertiary referral centre for CIED implantation and follow-up that serves a local population of 213 000 and the wider conurbation of Greater Manchester. This study included all adult patients (aged ≥ 18 years) with Triage-HFRS-enabled Medtronic CIEDs (defined as any CIED capable of measuring OptiVol™ 2.0 fluid-index) under follow-up between 21 June 2016 and 21 September 2018. The types of CIEDs we included were cardiac resynchronization therapy [CRT-D (with defibrillator), CRT-P (with pacemaker)], implantable cardioverter-defibrillators (ICDs) and pacemakers.

### Ethics

We applied to the United Kingdom (UK) Health Research Authority’s Confidentiality Advisory Group (CAG) to obtain a confidentiality waiver (Section 251) in the National Health Service Act to facilitate data linkage of specified patient-related data with Office of National Statistics (ONS) data from patients with a Triage-HFRS compatible CIED who were under follow-up at our institute between 21 June 2016 and 21 September 2018. This application was fully supported and in view of this a favourable opinion from the CAG was issued in May 2019 (19/CAG/0055). This study complies with the Declaration of Helsinki.

### Data sources and collection

Data for this study were acquired from three sources: hospital-based electronic health records at site (screening, demographic, and transmission data), Medtronic ‘CareLink’ network (transmission data), and NHS Digital (outcome data). CareLink is a Medtronic hosted cloud service that collates all Medtronic device transmissions globally (including hospital-based and home-based downloads). Local care providers access CareLink transmission data using the One Hospital Clinical Service portal, a medical care quality improvement platform which conforms to the principles outlined in the Declaration of Helsinki.

Linked mortality data were provided from NHS Digital (the national provider for NHS data collection), which collates data from all NHS providers in the UK to provide reliable and consistent information on hospital admissions and mortality. NHS Digital acquires its mortality data from the Office of National Statistics using information derived from death certificates. Given that registration of death is mandated in the UK, we regard such mortality outcome data to be robust and ensures that mortality data was available for all patients. The provided data included date of death and the leading cause-of-death.

A 6-digit patient pin was used to link pseudonymized outcomes data returned from NHS digital with Carelink and demographic data.

### CIED remote monitoring

Physiological parameter data were collected continuously by the device, and feeds into the Triage-HFRS algorithm (described elsewhere).^2^ Parameters vary slightly by type of device, as outlined in *Table 1*. The Triage-HFRS is calculated using the maximum measures from the previous 30 days, although daily risk is available using only the physiological data recorded in the preceding 24 h. The daily data are stored within the device until a transmission occurs [either an automated transmission triggered by detection of new AF, ventricular arrhythmia, high OptiVol™ (if activated), or a routine scheduled transmission undertaken every 3 months for most patients as per guideline recommendations].^11^ When a transmission is triggered, parameters for each day of recording are uploaded to the CareLink cloud where the HFRS is assigned. The classification of low-, medium-, or high-risk statuses is evaluated using the sum of the physiological parameters, across the relevant time window (30 days or daily, as relevant).

**Table 1:** ** **Heart Failure Risk Status (HFRS) parameters by device type

HFRS parameter	CRT-D	CRT-P	PPM
OptiVol™ (intrathoracic impedance)	✓	✓	✓
Physical activity	✓	✓	✓
Night ventricular rate	✓	✓	✓
Heart rate variability	✓	✓	✓
AF/atrial tachycardia (AT) burden	✓	✓	✓
Ventricular rate during AF/AT	✓	✓	✓
% CRT pacing	✓	✓	
Treated VT/VF	✓		
Shocks	✓		

AF, atrial fibrillation; CRT, cardiac resynchronization therapy; VT/VF, ventricular tachycardia/fibrillation.

### Outcomes

The observational primary endpoint of mortality was evaluated for all cases from the date of first transmission (on or after 18 April 2015), until either death occurred or the end of the evaluation period 06 November 2019. Causes of death were reviewed and categorized by two clinicians (F.Z.A. and J.K.T.).

### Statistical analysis

As exploratory analysis, we summarized temporal changes through daily low-, medium-, and high-risk statuses by considering the number of times each patient visited each status and the length of time they remained in that status before transitioning to a different status. To visualize the longitudinal time series data, we simulated hypothetical patient profiles of risk statuses through time, based on the observed data (see Supplementary material online, *Methods* for details).

For the purposes of the main analysis, we categorized patients into groups of either ‘high’ or ‘never-high’ based on their entire HFRS trajectory of daily statuses. Specifically, any patient evaluated to be at high risk at any point in their follow-up was categorized into the ‘high’ group, with all other patients forming the ‘never-high’ group. We chose this binary delineation *a priori* based on the clinical judgement and current perceptions that a medium status has so far not been shown to confer meaningful actionable information.

To investigate the association between patients with a high HFRS and mortality, we compared the mortality proportion of those in the ‘high’ group compared to the ‘never-high’ group using logistic regression. Similarly, we used logistic regression to evaluate the association between all daily risk status trajectory data and all-cause mortality. We describe the association using odds ratios (ORs) with corresponding 95% confidence intervals (CIs), where an OR >1 implies increased odds of mortality. Additionally, we compared time-to-death using Kaplan–Meier plots and log-rank tests across groups of ‘high’ or ‘never-high’; such groups were defined based on landmark times of 30, 90, 180, 365 days after study entry, where we condition on survival up-to the landmark time and define patients to the high group if they had at least one high HFRS prior to the landmark time.

Finally, we evaluated the predictive performance (in terms of calibration and discrimination) of the daily HFRS using established methods to quantify how well it performs at predicting mortality.^12^^,^^13^ Additionally, we considered model updating methods to revise the daily HFRS accounting for additional predictors that were not included in the original model (i.e. patient demographic data) to see if we could improve predictive performance.^14^^,^^15^ See Supplementary material online, *Methods* for details.

All analyses were performed using R version 3.6.1.^16^

## Results

### Study population

There were 439 patients under MHC follow-up during the study period (16 June 2016 to 21 September 2018). Of these, 167 (38%) had a CRT-D, 36 an ICD (8.2%), 172 (39.2%) a CRT-P, and 64 (14.6%) a pacemaker. Overall, 318 (73.3%) had a documented history of heart failure with reduced ejection fraction, and 243 (56.1%) had a last recorded left ventricular ejection fraction (LVEF) of ≤35%. Baseline clinical characteristics according to risk group are summarized in *Table 2*. Transmission data were available from 18 April 2015 (data stored on the device for up to 14 months prior to download).

**Table 2:** ** **Summary of patient demographics as a full cohort, and across groups of either never recorded high risk vs. at least one recorded high risk in follow-up

Demographics	Never recorded high (*n* = 154)	High (*n* = 285)	Total (*n* = 439)	*P*-value
Male, *n* (%)	96 (62.3%)	182 (63.9%)	278 (63.3%)	0.752
Age, mean (SD)	63.3 (15.6)	67.7 (15.2)	66.1 (15.5)	**0.004**
Index of multiple deprivation, median (IQR)	4 (2–7)	4 (1–7)	4 (2–7)	0.458
Known to HF team, *n* (%)	55 (35.7%)	117 (41.0%)	172 (39.2%)	0.274
History of atrial fibrillation/flutter, *n* (%)	58 (38.2%) Missing: 2	131 (46.1%) Missing: 1	189 (43.3%) Missing: 3	0.110
HF with reduced ejection fraction (HFrEF),^a^ *n* (%)	105 (68.6%) Missing: 1	213 (75.8%) Missing: 4	318 (73.3%) Missing: 5	0.108
Left ventricular ejection fraction <35%, *n* (%)	72 (47.4%) Missing: 2	171 (60.9%) Missing: 4	243 (56.1%) Missing: 6	**0.007**
Left ventricular ejection fraction <35% and known to HF team, *n* (%)	39 (54.1%)	91 (53.2%)	130 (53.5%)	0.892
Ischaemic heart disease, *n* (%)	82 (54.7%) Missing: 4	156 (55.9%) Missing: 6	238 (55.5%) Missing: 10	0.804
Adult congenital heart disease, *n* (%)	20 (13.2%) Missing: 3	19 (6.7%) Missing: 2	39 (9.0%) Missing: 5	**0.026**
Prior ablation,^b^ *n* (%)	15 (9.9%) Missing: 3	56 (19.9%) Missing: 4	71 (16.4%) Missing: 7	**0.009**
Prior myocardial infarction (MI), *n* (%)	55 (37.2%) Missing: 6	90 (32.6%) Missing: 9	141 (34.1%) Missing: 15	0.346
Chronic obstructive pulmonary disease (COPD), *n* (%)	14 (9.6%) Missing: 8	41 (14.9%) Missing: 9	55 (13.0%) Missing: 17	0.129
Diabetes, *n* (%)	28 (19.2%) Missing: 8	75 (27.3%) Missing: 10	103 (24.5%) Missing: 18	0.067
Chronic kidney disease stage (CKD) ≥3, *n* (%)	43 (27.9%)	92 (32.7%) Missing: 4	135 (31.0%) Missing: 4	0.299
Duration of follow-up (days), median (IQR)	538 (304–828)	765 (478–842)	702 (387–840)	**<0.001**
Device type, *n* (%)				0.378
CRT-D	58 (37.7%)	109 (38.2%)	167 (38.0%)	
CRT-P	53 (34.4%)	119 (41.8%)	172 (39.2%)	
ICD	18 (11.7%)	18 (6.3%)	36 (8.2%)	
PPM	25 (16.2%)	39 (13.7%)	64 (14.6%)	
NYHA Class, *n* (%)				**0.003**
No heart failure	30 (19.5%)	32 (11.2%)	62 (14.1%)	
1	27 (17.5%)	29 (10.2%)	56 (12.8%)	
2	51 (33.1%)	100 (35.1%)	151 (34.4%)	
3 or 4^c^	39 (25.3%)	108 (37.9%)	147 (33.5%)	
Not available (missing)	7 (4.5%)	16 (5.6%)	23 (5.2%)	
Medications, *n* (%)				
Beta blockers	108 (77.1%) Missing: 14	212 (80.9%) Missing: 23	320 (79.6%) Missing: 37	0.485
Ace-i/ARB/ARNI	99 (70.7%) Missing: 14	175 (67.3 %) Missing: 25	274 (68.5%) Missing: 39	0.557
MRA	40 (28.8%) Missing: 15	109 (41.9%) Missing: 25	149 (37.3%) Missing: 40	**0.013**
Diuretic	53 (38.1%) Missing: 15	153 (58.6%) Missing: 24	206 (51.5%) Missing: 39	**<0.001**

Boldface values indicate statistical significance at a glance.

Ace-i, angiotensin-converting enzyme inhibitor; ARB, angiotensin receptor blocker; ARNI, angiotensin receptor-neprilysin inhibitor; CRT-D, cardiac resynchronization therapy with defibrillator; CRT-P, cardiac resynchronization therapy with pacemaker; HF, heart failure; ICD, implantable cardioverter-defibrillator; IQR, interquartile range; MRA, mineralocorticoid-receptor antagonists; NYHA, New York Heart Association; PPM, pacemaker; SD, standard deviation.

a Recorded in electronic patient records.

b Includes any history of documented cardiac ablation for AT/AF or atrial flutter.

c Includes <5 patients with NYHA class 4 functional status.

### Risk status

CareLink data for 11 092 risk status periods were available. Of the 11 092 episodes, 4394 (39.6%) were low-, 5528 (49.8%) were medium-, and 1170 (10.5%) were high-risk statuses. The median number of days of transmitted data per patient during the 27-month follow-up was 703 days [interquartile range (IQR): 388–841].

**Figure 1:**
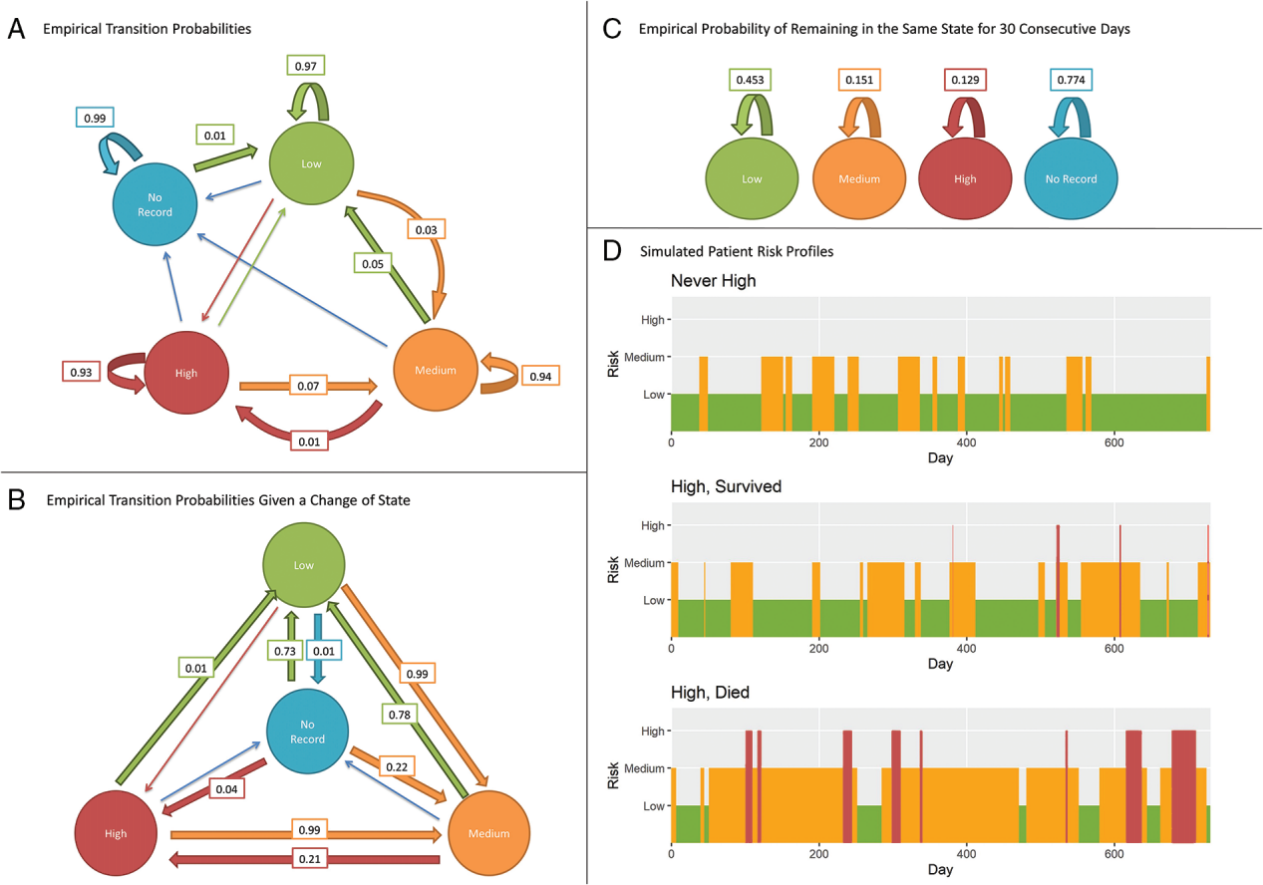
Empirical probabilities for (*A*) daily transitions, (*B*) transitions given that a change of status occurred, and (*C*) remaining in a risk status for 30 consecutive days. Arrows without probabilities have probabilities less than 0.005. (*D*) Simulated risk profiles over 2 years from entry, using empirical transition probabilities from each patient group.

The number of days spent in each low-, medium-, or high-risk status was recorded for each transmission. *Figure 1* demonstrates the empirical probabilities of daily transitions between risk status based on observed data, with example simulated risk profiles described in Supplementary material online. The average total number of days spent in each status is shown in *Table 3*. Overall, most time was spent in low-risk, followed by medium- and then high-risk. On average, a patient would spend 49 consecutive days in low-risk before transitioning to another risk status. Patients in the ‘never recorded high’ group spent between 68.4% and 90.5% of their follow-up in a low-risk status, and between 8.8% and 28.5% of their follow-up in a medium-risk status. Patients in the ‘high’ group who survived spent between 29.9% and 70.6% of their follow-up in a low-risk status, between 21.7% and 55.1% of their follow-up in medium-risk status, and between 1.3% and 10.2% of their follow-up in high-risk status (*Table 3*). In contrast, patients in the ‘high’ group who died spent a lower proportion of the evaluation period in a low-risk status compared to the other two groups, and a higher proportion of follow-up in a high-risk status. After 1 year of being monitored, over 50% of patients had entered high risk or been censored (Supplementary material online, *Figure* *S1*).

**Table 3:** ** **Average time spent in each status for patients with at least one transmitted HFRS in that status

	Low	Medium	High	No record (between first and last recorded risk)
Never recorded high (*N* = 154)				
Total days	465.5	88.5	–	185.9
Number of consecutive days to change	72.0	13.5	–	124.5
IQR Proportion of Follow-up	68.4–90.5%	8.8–28.5%	–	0.0–0.0%
High, survived (*N* = 236)				
Total days	367.6	259.4	54.5	135.1
Number of consecutive days to change	37.9	25.3	13.6	123.7
IQR proportion of follow-up	29.9–70.6%	21.7–55.1%	1.3–10.2%	0.0–0.0%
High, died (*N* = 50)				
Total days	190.2	271.6	97.1	167.0
Number of consecutive days to change	25.7	35.2	23.7	83.5
IQR proportion of follow-up	1.9–51.0%	33.7–66.0%	5.3–25.6%	0.0–0.0%
All (*N* = 440)				
Total days	384.1	201.1	61.9	147.2
Number of consecutive days to change	48.8	22.3	15.4	122.8
IQR proportion of follow-up	33.7–82.3%	15.1–51.1%	0.0–7.2%	0.0–0.0%

IQR, interquartile range.

### Mortality

In total, 60 patients died during follow-up. The maximum recorded risk status before death was either medium (*n* = 10) or high (*n* = 50), meaning that no patients whose risk status remained low for the duration died. There was often a missing period of HFRS data leading up to death, hence it may be that patients with a medium maximum recorded risk experienced a high-risk status in the lead up to death but it was not transmitted (e.g. hospitalization resulting in loss of transmission of HFRS data to CareLink). The median time between the last transmitted HFRS and death was 111 days (IQR: 57–226) and the median time from the last maximum recorded risk and death was 233 days (IQR: 91–390). Of those that died, 53 had a CRT device (26 had a CRT-D, 27 CRT-P) and 7 had non-CRT devices.

Of the 60 deaths, 35 were cardiovascular deaths. Causes of death included (in descending order of frequency): cardiovascular disease (*n* = 35, of which HF accounted for 6 deaths), respiratory disease (*n* = 7), cancer (*n* = 6), renal failure (*n* < 5), falls (*n* < 5), diabetes (*n* < 5), and dementia (*n* < 5). In 5 cases the cause of death was missing. There were significantly more cardiovascular deaths in the high group, compared to the never-high group (10.3% vs. <4.0%; *P* = 0.03).

**Figure 2:**
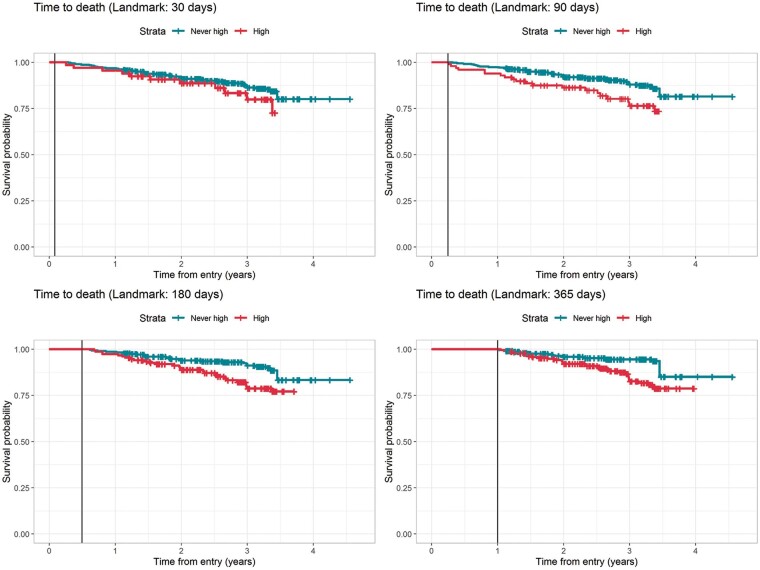
Kaplan–Meier survival curves with landmarks at 30, 90, 180, and 365 days determining ‘baseline’ group assignment to ‘Never recorded high’ or ‘High’.

The odds of all-cause mortality were significantly higher in patients with at least one high-HFRS, irrespective of the duration of said high, compared to patients who were not recorded as having a high-HFRS during their follow-up (OR: 3.07, 95% CI: 1.57–6.58, *P* = 0.002). Similar findings were observed for time-to-death; survival was significantly worse in the ‘high’ group compared with the ‘never-high’ group, across all landmark times considered (see Methods and *Figure 2*).

**Figure 3:**
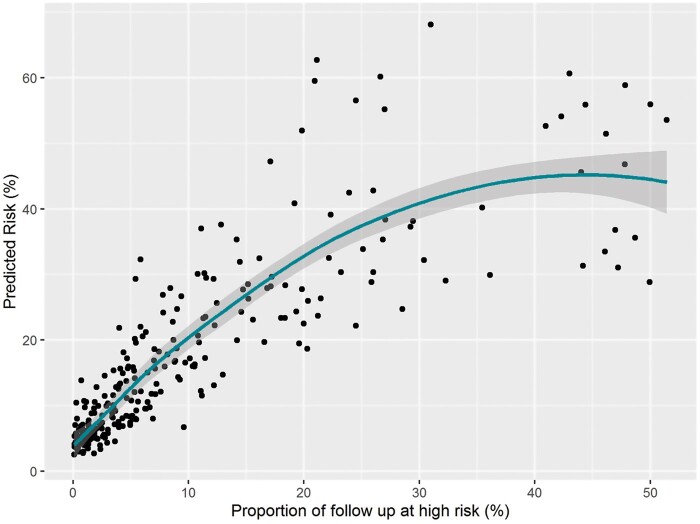
Overall predicted risk of death after adjusting for the proportion of time recorded in each status from the logistic regression model described in *Table 4*.

In patients that experienced at least one high, the time spent in high-risk status was, on average, 42.6 days longer for those that died compared to those who survived (*P* = 0.002). The percentage of the follow-up that was spent in high was also shown to be associated with death (Supplementary material online, *Table* *S2*), after adjusting for the length of follow-up and proportion of time spent in low risk. The corresponding predicted risks are shown in *Figure 3*. In addition, when we considered only the high-risk episodes which lasted at least 14 consecutive days as a predictor, we found that each of these was associated with increased odds of death (OR: 1.26, 95% CI: 1.06–1.48; *P* = 0.006). Sensitivity analyses using an HF sub-population [CRT device or New York Heart Association (NYHA) 2+ or LVEF < 35] demonstrated that the odds ratio remained statistically significant and was similar for the high HFRS (OR: 2.33, 95% CI: 1.15–5.13, *P=* 0.026) (Supplementary material online, *Table* *S3*). Similar findings were found when we adjusted for NYHA and CRT device (Supplementary material online, *Table* *S4*).

### Predicted risk of mortality after adjusting for time spent in each state

**Figure 4:**
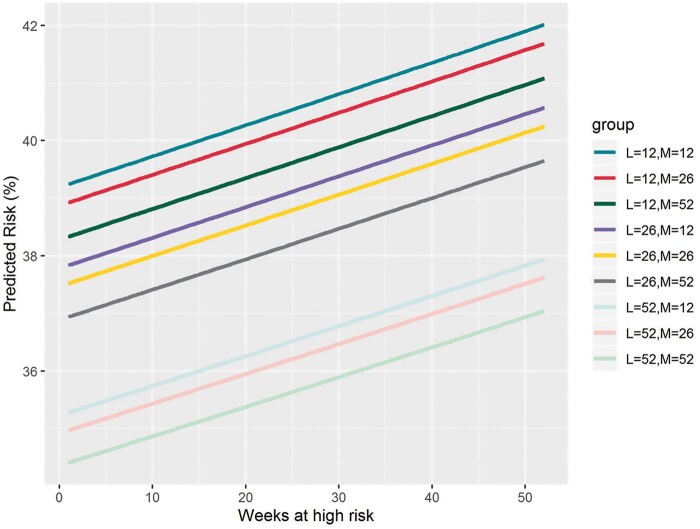
Predicted risk of mortality after adjusting for time in each risk status. The predicted risk of mortality after adjusting for time spent in each state suggests that a patient who spent 26 weeks in ‘low’, 52 weeks in ‘medium’ (dark grey line), and approximately 2 weeks in ‘high’ had the same predicted risk of death as a patient that spent 52 weeks in ‘low’, 12 weeks in ‘medium’ (light blue line), and approximately 34 weeks in ‘high’, with a risk of 37%. Similarly, a patient that spent 12 weeks in ‘low’, 12 weeks in ‘medium’ (mid-green line), and 15 weeks in ‘high’ (*x*-axis) had the same risk of death as a patient that spent 12 weeks in ‘low’, 26 weeks in ‘medium’ (red line), and 21 weeks in ‘high’ (*x*-axis), with a risk of 40% (*y*-axis).

An increased number of days spent in a high-risk state was associated with significantly increased mortality (OR: 1.00, 95% CI: 1.00–1.02, per day; *P* = 0.015). Similarly, a decreased number of days spent in a low-risk state was associated with significantly increased mortality (OR: 1.00, 95% CI: 0.99–1.00, per day; *P* < 0.001), after adjusting for recorded time spent in each state (Supplementary material online, *Table* *S1*). The predicted risk of mortality after adjusting for time spent in each state is outlined in *Figure 4*.

### Improving identification of patients at increased risk of death

To explore if the addition of demographic factors to the daily HFRS could improve prediction of risk of death, we added Age, Gender, LVEF ≤ 35 and chronic kidney disease (CKD) stage ≥ 3 into the daily HFRS (only these were considered due to the small number of events), as shown in *Table 4*. Including these additional predictors significantly improved the predictive performance of the model, increasing the optimism-adjusted area under the curve (AUC) from 0.61 (95% CI: 0.56–0.66) for the original HFRS model to 0.72 (95% CI: 0.67–0.78) for the updated HFRS model. Furthermore, the optimism-adjusted calibration of the updated HFRS model was superior to that of the original HFRS model (Supplementary material online, *Table* *S5*).

**Table 4:** ** **Multivariable Logistic regression and lasso logistic regression model coefficients (*n* = 431), with the percentage of times a predictor was selected using the lasso procedure in the bootstrap internal validation procedure (*N* = 1000)

Predictor	Logistic regression odds ratio	95% CI	*P*-value	Lasso regression odds ratio	% occurrences
Intercept	0.00	0.00–0.01	**<0.001**	0.00	100
Age	1.05	1.02–1.08	**<0.001**	1.05	100
Male	1.39	0.74– 2.67	0.311	1.32	72.4
CKD ≥stage 3	2.08	1.14–3.81	**0.017**	1.91	96.4
High ≥1	2.43	1.20–5.33	**0.018**	2.19	98.7
LVEF < 35	1.58	0.83–3.14	0.174	1.56	88.6

Boldface values indicate statistical significance at a glance.

See Supplementary material online, *Methods* for details.

CI, confidence interval; CKD, chronic kidney disease; LVEF, left ventricular ejection fraction.

## Discussion

This study reports that HFRS data can be used to predict all-cause mortality in patients with a full range of CIEDs and differing indications. Specifically, routinely monitored data, which is automatically collected on a daily basis as part of the Medtronic Triage-HFRS clinical tool, helped discriminate patients both at high and low risk of death. Moreover, there was a three-fold increased odds for patients who spent at least one day in the high HFRS, with a 26% increase in odds of mortality for patients who had 14 consecutive days or more in a high-risk status. Patients who experienced a high HFRS and died spent significantly higher percentages of time in a high-risk status, and less time in a low-risk status, compared to those that did not die. On the other hand, being in a persistent low-risk status throughout the follow-up period resulted in no deaths.

This study is part of a larger body of work looking at how remotely monitored CIED physiological data and the HFRS can be used to predict adverse outcomes, and risk stratify at an individual patient level to guide monitoring programmes.

### All-cause mortality

In the current analysis, the CRT population had the highest proportion of deaths and high HFRS events. In view of this, we conducted a sensitivity analysis adjusting for device type in the HF population. This analysis reported a consistent estimated odds ratio for a high HFRS event across both the HF and unselected population.

It was theorized that physiological parameters from CIED data would predict all-cause mortality. Individual parameters such as heart rate, rhythm and physical activity are all known to confer prognostic information for patients with and without HF.^1^^,^^17–21^ The cohort of patients in this study are diverse—ranging from young patients with primary prevention ICDs, to older multimorbid HF cases. Causes of death were also diverse, but cardiovascular death was the most common mode of death. Although there were only a small number of cardiovascular deaths, significantly more were observed in the high group, with mortality rates across groups of high vs. never-high 10.3% vs. <4.0%; *P* = 0.03. We were unable to investigate predictive performance for estimating cardiovascular-related mortality due to the small number of cardiovascular deaths, as a consequence of the sample size. With respect to non-cardiovascular deaths, it would also be reasonable to expect heart rate and thoracic impedance be altered in any patient with severe respiratory illness or advanced systemic disease with end-stage cardiopulmonary complications. Rapid changes in physiological parameters may also indicate disease instability—this would require further investigation. Furthermore, although the current study examined the utility of the HFRS for the remote identification of patients at high risk of mortality, it should be noted that primary use of this technology is for the early detection of individuals at increased risk of HFH, alerting patient care teams to the potential risk of deterioration. We have previously reported the utility of the rich physiological data provided by components of the risk score to complement phone-call based consultations and guide interventions.^4^ However, further work is needed to understand the specific pathophysiology during high-risk states and what interventions, if any, can mitigate patient clinical deterioration or support end-of-life management.

### Prognostic risk scores in clinical practice

Estimation of prognostic outlook in patients with CIEDs is important for a number of reasons, for example to guide discussions of appropriateness of therapies and decisions around clinical care and escalation. Despite this, use of prognostic risk scores in clinical practice is limited.^5^

Several prognostic scores have been developed or adapted for use in patients with CIEDs. Many of these scores such as MADIT-II, FADES, PACE, SHOCKED, CHADS_2_, and CHADSVASC^5^ have limited use in practice for several reasons.^6^^,^^7^^,^^21^ First, for the most part, these scores have not been externally validated in large-scale studies. Second, some of the scores were developed for use in highly selective device populations (CRT or ICD recipients), limiting their scope of use to the wider CIED cohort. Third, calculation of scores and determination of risk can be labour intensive for use in routine clinical practice with lack of automation. Fourth, the dynamic nature of important variables such as renal function or brain natriuretic peptide are not considered across the clinical time-course and finally, none of these scores utilize the rich real-time physiological data made available by the CIED.

Various multivariable risk scores for the prediction of mortality in HF, where use of CIEDs is commonplace, have also been produced. However, the routine use of these risk tools (such as Seattle Heart Failure Model and MAGGIC Risk Score) in clinical practice is not commonplace.^8^^,^^9^ Again, key to ensuring optimum performance is foreknowledge of numerous clinical variables, many of which vary over time and are not routinely measured or recorded in an ambulatory device population (e.g. uric acid, cholesterol levels, sodium, medication doses, current smoking status, body mass index), making these risk scores cumbersome for use within the constraints of usual clinical practice.^8^^,^^9^^,^^21^

Triage-HFRS differs from previous scores in this respect and is unique in that it leverages remote monitoring data, routinely collected by sensors within the implanted device, for prognostic purposes. The system is fully automated to compute a personalized risk of a future event; this goes beyond its original use case scenario of identifying patients at risk of a HFH within 30 days.^1^^,^^2^ This novel automated approach to data collection has distinct advantages over the limitations of existing scores.

Triage-HFRS is weighted by the OptiVol™ index. Previous studies have demonstrated links between both OptiVol™ crossing and absolute impedance and mortality in patients with devices that are capable of remote monitoring.^22^^,^^23^ Subsequent to this multiple studies have demonstrated the incremental value of multi-sensor algorithms over single parameter monitoring.^1^^,^^2^ However, for the most part these studies have been limited to predicting risk of HFH. In this evaluation, we have shown that an easy to use Triage-HF risk-stratification tool also had good predictive value for mortality. Future studies should compare the performance of these different prognostic classifications to determine which has the best association with mortality. Consistent with our findings, a recent large retrospective analysis of US Healthcare claims data reported that a high risk status was associated with a 4-year all-cause mortality risk of 38%.^24^

In the current analysis, we report that the predictive performance of the ‘high’ Triage-HFRS to identify patients at increased odds of mortality can be augmented by knowledge of age, gender, and CKD status. Interestingly, removing LVEF ≤35% from the model did not alter the AUC (0.72). Since age and gender are a known entity, and the high-risk status is provided by the device, the only additional clinical data required to utilize this improved model is knowledge of whether estimated glomerular filtration rate is <60 mL/min. Hence, this makes the updated model universally easy to adopt into clinical practice. Furthermore, with an AUC of 0.72 the augmented Triage-HFRS is at the very least, comparable with existing clinical risk scores used to predict mortality in patients with HF—but less labour-intensive, with lower risk of having missing data points.^21^ It should be noted that the HFRS examined in the current manuscript is only available for compatible Medtronic devices and therefore application of this technology is limited to these populations. However, other manufacturers have developed their own HF management tools. Future studies examining the performance of these risk tools and solutions which address the issue of hosting multivendor platforms are desirable.

### Implication for clinical practice

**Figure 5:**
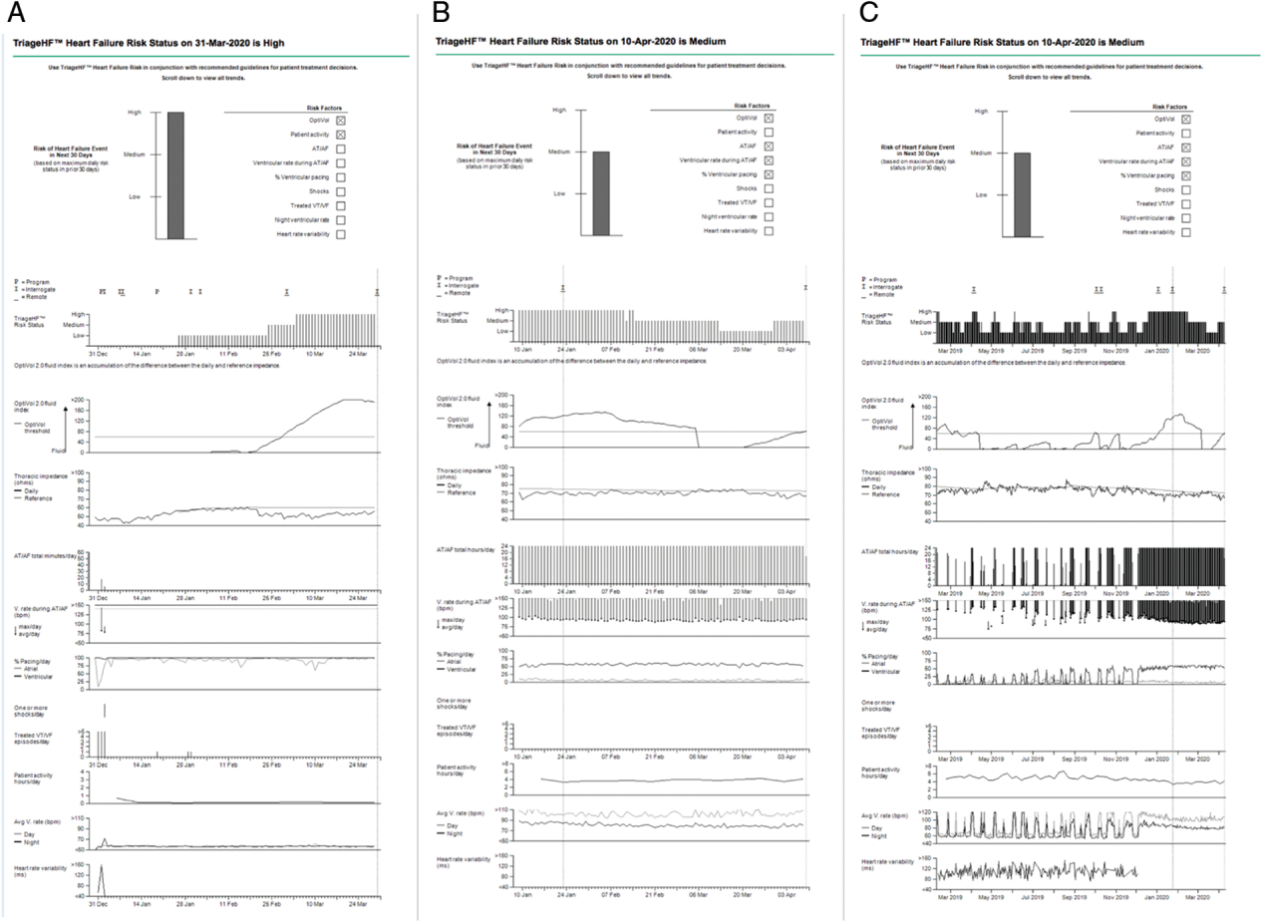
Clinical management report derived from the CIED. Individual physiological data (comprised of OptiVol™ 2.0 fluid-index, thoracic impedance, activity, daily AF burden, etc.) are updated daily and combined to compute the Triage-HFRS. Individual parameters significantly contributing to the current Triage-HFRS are indicated by the checked boxes (top). (*A*) Corresponding with a fall in thoracic impedance and a rise in the OptiVol™ 2.0 fluid-index, this 90-day zoom depicts the HFRS transitioning from Low-, to Medium-, and then ultimately High-risk status. Each vertical line in the HFRS (90-day zoom) depicts 1 day. Of note, the Medium-risk status appear to be a transitionary risk state from which the patient moves up to High, or down to a Low risk status. (*B*) A 90-day zoom report showing a High HFRS in January/February, with subsequent transition to a Medium-risk status. (*C*) 14-month trended data for the same patient presented in *B*.

The Triage-HFRS is available for clinicians to use as part of standard practice since reporting is a standard output within the clinical management report (*Figure 5*). The combined Triage HFRS algorithm was designed to predict impending decompensated HF episodes, and thus in its current format is designed to monitor HF stability. Previous studies have reported the utility of the HFRS to identify patients at increased risk of HFH, and have restricted the scope of research to just those patients with CRT-D or high power devices. The current study expands on this and examines the relationship between the HFRS and mortality. Our study opens up avenues for monitoring patient’s medium- to long-term mortality risk based on their remote monitoring data. This may focus the practitioner’s attention to ensuring that the patient is in receipt of guideline-directed therapies designed to improve long-term prognosis rather than avert an immediate crisis. In addition—given association with all-cause mortality—this may trigger the practitioner to take a more holistic approach to their assessment. Personalized information may also help clinicians make decisions regarding frequency of monitoring, for example there may be justification in reducing surveillance for low-risk individuals. These areas of clinical application require further research.

Finally, our study also highlights that routine remote monitoring of patients with CIEDs could act as a failsafe: minimizing the risk of patients with significant cardiac disease being lost to follow-up. For example, in the current study, it was found that almost half of all patients with a high HFRS and LVEF <35% were not known to the HF team for regular follow-up.

### Limitations

Patients who had opted out of the national data collection scheme (required to access NHS Digital mortality data) were not included in the current analysis. However, on the basis that nationally very few patients opt out of this scheme we envisage this to have had minimal impact on the current analysis.

The current manuscript examines the utility of the device-derived Triage-HFRS as a stand-alone tool to risk-stratify at a population level and identify individuals at high risk of death. Although the current study did not consider any downstream human interaction out with usual standard of care, various service improvement projects were taking place during the study period. Individuals in the current analysis had been included in the previous Triage-HF Plus evaluation.^4^ When all cases are considered, this represents at most 90/11 092 (0.8%) episodes examined in the current study, which is unlikely to have impact on the outcomes observed in this study.

Triage HFRS is available across CRT and non-CRT devices. In view of this, the parameters which feed into the HFRS may differ, not only according to device type but also between patients; for example, assessment of ventricular tachycardia therapies for high power devices, those with and without AF (HRV vs. No HRV data), or those with or without an atrial lead (differential reporting on AF parameters). However, since this is the first publication to examine Triage HFRS across the full spectrum of devices (CRT and non-CRT) there is as yet no published data on the differential performance of the HFRS between device types and populations—but this is area on ongoing future work.

Periods without transmitted data are encountered in clinical practice, as was observed in 36 patients within the current evaluation (episodes: 45; median length: 65 days). They are often attributable to just a few key reasons. First, if a patient fails to record a transmission within a 425 days window, data will be lost. Second, post-implant there is a run-in period of 65 days where CareLink withholds the HF risk status (34 days to allow for pocket maturation for OptiVol impedance and a further 30 days to collect impedance data for the HF risk algorithm). Third, if the device is permanently unpaired from the CareLink monitor (e.g. patients who die in hospital or are discharged to accommodation without their home monitor), no data are transmitted hence missing transmission data are common in the lead up to death. Of relevance, in the current analysis, ten patients who died never recorded a high-risk status. A proportion of these patients died in hospital (median length of stay 24 days). Therefore, although the highest/last transmitted risk status prior to death recorded by CareLink was ‘medium’ it is entirely feasible that a higher risk status could have been recorded during the period of hospitalization. The impact of missing data from patients hospitalized close to death is unclear. Finally, the current analysis utilized the primary leading cause of death. Whether a cardiovascular condition contributed to the death was not examined. A repeat analysis examining cardiovascular deaths as the main outcome is recommended, but the current study was not powered to examine this. Therefore, we propose further work, in larger populations, that aims to specifically explore the predictive ability of the HFRS on risk of cardiovascular death.

## Conclusions

Remote monitoring data from CIEDs can identify patients at high risk of all-cause mortality, which is important, as anticipated life expectancy impacts upon clinical decision-making.

## Supplementary material

Supplementary material is available at *Europace* online.

## Supplementary Material

euab160_Supplementary_Data
