# Supplementary material for: Remote monitoring data from cardiac implantable electronic devices predicts all-cause mortality
Source: Europace. 2021 Oct 3;24(2):245–55. doi: 10.1093/europace/euab160 (PMC8824524; doi:10.1093/europace/euab160)
Supplement: euab160_Supplementary_Data [file euab160_supplementary_data.zip › euab160-suppl_data/Supplementary Data_08_06_2021_clean.docx]

# Supplementary statistical methodology

Descriptive statistics were produced to compare the never-high population to those who experienced at least one high measurement. Continuous variables are presented using the mean and standard deviation, or the median and interquartile range, as appropriate. Categorical variables are presented using counts and percentages.

Profiles of risk were simulated for the ‘never recorded high’, ‘high, survived’ and ‘high, died’ groups of patients. Let denote the risk state on day and for each of the patient groups, , let denote the empirical transition probability distribution. To generate the profiles, we followed the following procedure: 1) Evaluate the empirical distribution of the first risk state. 2) Generate a random value, , from the uniform distribution U(0,1); if  *≤* (=low), set =‘low’, if (=low) < ≤ (=low)+(=medium), set =‘medium’, otherwise set =‘high’. 3) For *i* = 1,…,730, repeat: given risk at day *i-1*is , where is either ‘low’, ‘medium’ or ‘high’, find the empirical probabilities of transitioning from that state to ‘low’, ‘medium’ or ‘high’, i.e. (low | ), (medium | ), (high | ). Generate a random value, , from the uniform distribution U(0,1); if  *≤* (low | ), set “low”, ); if (low | )*≤*  *≤* (low | )+ (medium | ), set “medium”, otherwise set “high”.

**Probability of daily transitions between risk score**

**Figure 1a** demonstrates the empirical probabilities of daily transitions between risk statuses. For example, if a patient was observed to be low-risk on a given day, then their probability of remaining low-risk the next day was 0.97 and their probability of transitioning to medium-risk was 0.03. When a change in risk status occurred, a patient that was in high- or low-risk would almost always move to medium-risk (**Figure 1b**). However, if the patient was in medium-risk, there was less certainty as to which status they would transition to, with 78.3% moving to low-risk and 21.4% moving to high-risk (**Figure 1b**). The probabilities for remaining in a low-, medium- or high-risk status for 30 consecutive days were 0.453, 0.151 and 0.129, respectively (**Figure 1c**). The empirical probabilities were also found within the groups ‘never recorded high’, ‘high, survived’ and ‘high, died’. These probabilities were used to generate the simulated risk profiles (**Figure 1d**), as described in the supplementary methods.

**Logistic regression methodology**

Specifically, we fit a logistic regression model under a penalised Lasso likelihood, where mortality was the outcome and with the HFRS and the new predictors (patient demographic data) as covariates. The predictive performance of the original and updated HFRS was quantified using calibration (agreement between the observed and expected event rate) and discrimination (ability of the model to differentiate cases from controls). Calibration was quantified using the calibration-in-the-large and calibration slope, while discrimination was quantified using the area under the receiver operating characteristic curve (AUC). Such performance metrics were assessed using bootstrap internal validation (N=1000) to correct for in-sample optimism.

**Supplementary Figures and Tables**

**Supplementary Figure 1: Empirical probabilities for a) daily transitions, b) transitions given that a change of status occurred, and c) remaining in a risk status for 30 consecutive days.** Arrows without probabilities have probabilities less than 0.005. **d) Simulated risk profiles over 2 years from entry, using empirical transition probabilities from each patient group.**

**Supplementary Figure 1: Kaplan-Meier curve demonstrating the time to the first high risk event.**


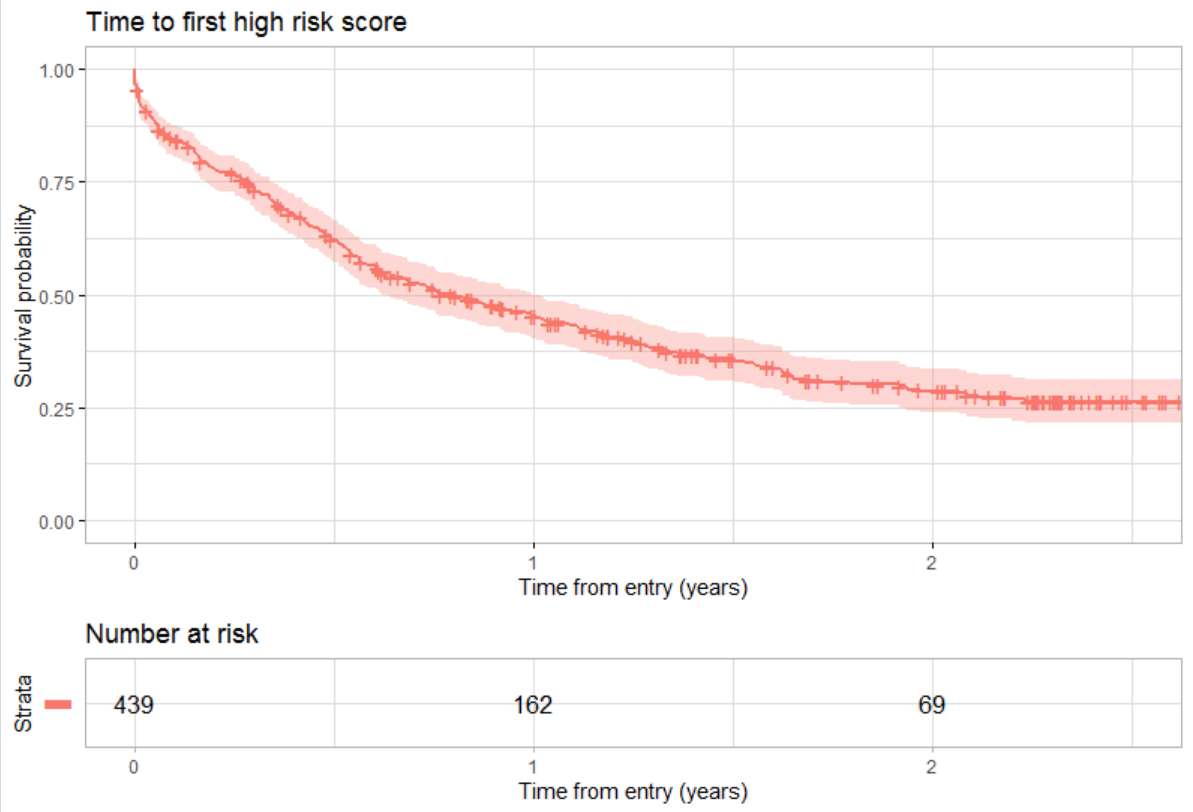


**Supplementary Table 1: Odds ratios for weeks spent in each state associated with mortality.**

| Predictor | Odds Ratio | 95% CI | p |
| --- | --- | --- | --- |
| Number of weeks at “low” | 0.97 | 0.96 - 0.98 | **<0.001** |
| Number of weeks at “medium” | 0.99 | 0.98 - 1.00 | 0.087 |
| Number of weeks at “high” | 1.10 | 1.03 - 1.19 | **0.015** |
| (Number of weeks at “high”)^2 | 1.00 | 1.00 - 1.00 | 0.068 |

**Supplementary Table 2: Odds ratios for proportion of time recorded in each state associated with mortality.**

| Predictor | Odds Ratio | 95% CI | p |
| --- | --- | --- | --- |
| Intercept | 0.33 | 0.08 - 1.28 | 0.113 |
| Percentage of FU at “low” | 0.99 | 0.97 - 1.01 | 0.180 |
| Percentage of FU at “high” | 1.13 | 1.03 - 1.24 | **0.007** |
| (Percentage of FU at “high”)^2 | 1.00 | 1.00 - 1.00 | **0.036** |
| Length of FU (years) | 0.59 | 0.37 - 0.92 | **0.021** |

**Supplementary Table 3: Sensitivity analysis multivariable logistic regression (n=370)** **using only the heart failure sub-population, i.e. CRT device or NYHA 2+ or LVEF<35.**

| **Predictor** | **Logistic Regression Odds Ratio** | **95% CI** | **p** |
| --- | --- | --- | --- |
| Intercept | 0.00 | 0.00 – 0.02 | **<0.001** |
| Age | 1.05 | 1.02 – 1.08 | **0.003** |
| Male | 1.49 | 0.78– 2.92 | 0.236 |
| CKD>=stage 3 | 2.04 | 1.11 – 3.76 | **0.021** |
| High>=1 | 2.33 | 1.15 – 5.13 | **0.026** |
| LVEF<35 | 1.44 | 0.73 – 2.97 | 0.302 |

**Supplementary Table 4: Sensitivity analysis multivariable logistic regression (n=354) adjusting for device type (CRT vs non-CRT) and NYHA (baseline NYHA=1) on all patients with NYHA 1+ (n=354).**

| **Predictor** | **Logistic Regression Odds Ratio** | **95% CI** | **p** |
| --- | --- | --- | --- |
| Intercept | 0.00 | 0.00 – 0.01 | **<0.001** |
| Age | 1.06 | 1.03 – 1.09 | **0.001** |
| High>=1 | 2.22 | 1.09 – 4.90 | **0.036** |
| CRT device | 1.13 | 0.42 – 3.57 | 0.824 |
| NYHA=2 vs NYHA=1 | 1.38 | 0.47 – 5.10 | 0.586 |
| NYHA>=3 vs NYHA=1 | 2.34 | 0.81 – 8.55 | 0.146 |

**Supplementary Table 5: Model performance measures of original HFRS model (high as only predictor) and the updated HFRS estimated by Lasso penalised regression.**

| **Optimism Adjusted Measure** | **Original HFRS Model** | **Updated HFRS*** |
| --- | --- | --- |
| AUC | 0.61  (0.56 – 0.66) | 0.72  (0.67 – 0.78) |
| Calibration in the large | 0.15  (-0.74 – 2.09) | 0.04  (-0.41 – 0.72) |
| Calibration slope | 1.08  (0.57 – 2.07) | 1.01  (0.72 – 1.40) |
