# Supplementary figures and images for: Remote monitoring data from cardiac implantable electronic devices predicts all-cause mortality
Source: Europace. 2021 Oct 3;24(2):245–55. doi: 10.1093/europace/euab160 (PMC8824524; doi:10.1093/europace/euab160)

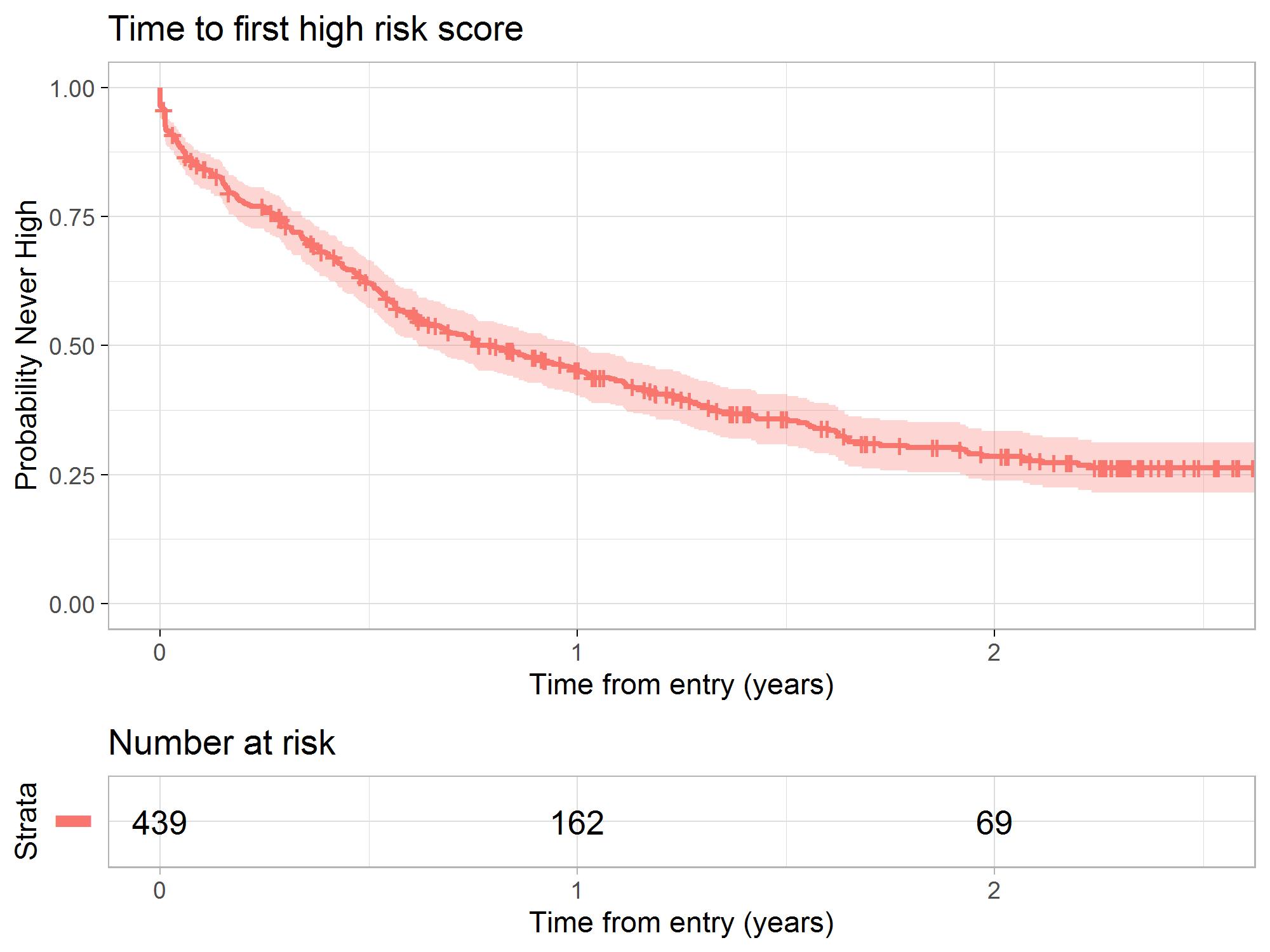

Supplement: euab160_Supplementary_Data [file euab160_supplementary_data.zip › euab160-suppl_data/Fig S1. km_time_to_high.jpeg]
